# Topology‐Driven X‐Shaped DNA as a cGAS Stimulator to Promote Dendritic Cell Activation and Antitumor Vaccine Efficacy

**DOI:** 10.1002/advs.78563

**Published:** 2026-10-29

**Authors:** Jiabei Li, Xuan Wang, Dali Wei, Zhejie Chen, Ziyi Wang, Qing Yu, Yu Yang, Shengpeng Wang

**Affiliations:** ^1^ State Key Laboratory of Mechanism and Quality of Chinese Medicine University of Macau Macao SAR China; ^2^ Institute of Molecular Medicine (IMM) School of Medicine Renji Hospital Shanghai Jiao Tong University Shanghai China

**Keywords:** cancer immunotherapy, cGAS‐STING signaling, DNA nanotechnology, DNA topology, vaccine adjuvants

## Abstract

Vaccine adjuvants are essential for enhancing immune responses, yet the development of safe and potent adjuvants remains challenging. Here, we report a topology‐guided nucleic acid adjuvant based on a four‐way Holliday junction framework (X‐DNA) designed to potentiate cGAS‐STING signaling. In contrast to linear dsDNA, X‐DNA presents a flexible, multibranched architecture that increases local DNA density and multivalent interaction potential, thereby favoring productive cGAS assembly. Molecular dynamics simulations demonstrate that X‐DNA forms a more stable complex with cGAS and facilitates the establishment of the active 2:2 cGAS‐DNA core assembly. Consistent with this mechanism, X‐DNA markedly enhances cGAMP production and IFN‐β secretion, promotes dendritic cell maturation and antigen presentation, and, when conjugated with antigenic peptides, strengthens antigen‐specific CD8^+^ T‐cell responses in vivo. In murine tumor models, X‐DNA significantly improves the therapeutic efficacy of peptide vaccination and suppresses tumor progression. These findings identify DNA topology as a programmable determinant of cGAS activation and establish X‐DNA as a structurally defined nucleic acid adjuvant for enhancing antitumor subunit vaccine efficacy.

## Graphical abstract

X‐shaped DNA promotes multivalent cGAS assembly and enhances cGAS‐STING signaling. When conjugated with antigenic peptides, the resulting X‐DNA‐based peptide vaccine promotes dendritic cell activation, antigen presentation, and antitumor T‐cell responses. These findings highlight the potential of programmable DNA structure design in the development of more effective and structurally defined antitumor peptide vaccines.

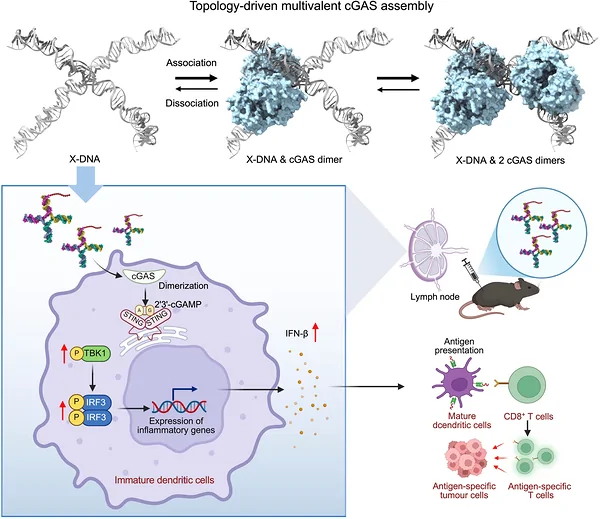

## 1 Introduction

Subunit vaccines have been widely applied in infectious disease prevention and tumor immunotherapy due to their safety and controllability profiles [1, 2, 3]. However, their limited capacity to elicit innate immune activation often requires the use of adjuvants to enhance immunogenicity [4, 5, 6, 7]. Contemporary vaccine adjuvants primarily promote antigen‐presenting cells (APCs) maturation and antigen presentation by activating host pattern recognition receptors (PRRs) [7, 8]. Among these, nucleic acid‐sensing PRRs play a particularly prominent role: TLR9 recognizes CpG DNA [9], RIG‐I detects 5’‐ppp RNA [10], and TLR3 senses double‐stranded RNA [11], triggering downstream type I interferon and inflammatory signaling pathways [7]. Notably, the cyclic GMP‐AMP synthase‐stimulator of interferon genes (cGAS‐STING) axis, a cytosolic DNA‐sensing pathway, induces potent type I interferon responses and has emerged as a core target for tumor immunotherapy and next‐generation adjuvant development.

The programmable sequence and structural versatility of nucleic acids enable precise regulation of innate immune signaling. Recent studies have shown that diverse nucleic acid topologies and delivery strategies can markedly enhance the cGAS‐STING signaling cascade. For example, Y‐shaped DNA structures [12] and hairpin oligonucleotides (Svg3) [13] directly augment cGAS enzymatic activity; three‐dimensional DNA frameworks such as DNA tetrahedra present immunostimulatory DNA (ISD) in a multivalent fashion [14]; and rolling‐circle amplification (RCA) products self‐assemble into virus‐like particles that, through dense spatial organization, induce cGAS phase separation [15]. In parallel, responsive and programmable architectures have further expanded the utility of nucleic‐acid adjuvants: miR‐21‐triggered DNA cascade systems enable tumor microenvironment‐selective cGAS activation [16, 17, 18], while modular DNA nanodevices (DNDAs) achieve targeted delivery of short Y‐shaped DNA fragments to enhance macrophage‐mediated signaling [19]. Taken together, these studies establish programmable nucleic‐acid architectures as a versatile platform for rational manipulation of innate immune recognition.

Structural biology studies have elucidated the fundamental principles governing cGAS‐dsDNA assembly. Two cGAS monomers cooperatively bind dsDNA to form a 2:2 cGAS‐DNA complex, which represents the minimal catalytic unit required for cGAMP synthesis [20, 21, 22]. This complex preferentially adopts a parallel DNA alignment with an interhelical spacing of ∼1 nm [23] and recognizes the DNA backbone in a sequence‐independent manner [21, 24, 25]. cGAS activation is highly sensitive to DNA length, with human cGAS typically requiring DNA fragments longer than ∼45 bp to support stable oligomerization and cooperative assembly [20, 26]. On extended DNA substrates, cGAS can further organize into ladder‐like higher‐order networks [27]. DNA‐bending proteins such as TFAM and HMGB1 further facilitate cGAS nucleation by inducing sharp DNA curvature [27], while multivalent cGAS‐DNA interactions promote liquid‐liquid phase separation, forming condensates enriched in cGAS and DNA that amplify downstream signaling [17, 28]. These structural insights highlight the critical role of DNA topology, multivalency, and spatial organization in regulating cGAS activation.

Guided by these principles, we designed an X‐shaped framework nucleic acid (X‐DNA) featuring a flexible, multibranched geometry. X‐DNA consists of four interconnected double‐stranded arms arranged in a Holliday junction‐like configuration, in which the central junction provides tunable bending angles and multivalent proximity clustering. Such architecture generates high local density and multivalent binding interfaces, thereby enabling efficient three‐dimensional recruitment and stabilization of cGAS. Molecular docking and dynamics simulations further suggest that X‐DNA stabilizes cGAS‐DNA assemblies and supports multivalent cGAS engagement. Functionally, X‐DNA markedly enhances intracellular cGAMP and IFN‐β production, upregulates CD80/CD86 expression on dendritic cells (DCs), and promotes antigen presentation. When co‐administered with antigenic peptides, X‐DNA robustly amplifies antigen‐specific CD8^+^ T‐cell responses and suppresses tumor growth in mice. Collectively, X‐DNA exploits engineered topology to recapitulate and reinforce the structurally driven assembly of cGAS, establishing a programmable structural nucleic‐acid adjuvant strategy for subunit vaccines and providing a mechanistic framework for the application of DNA nanostructures in tumor immunotherapy.

## 2 Results and Discussion

### 2.1 X‐DNA Engages cGAS and Enhances cGAMP Production

As a cytosolic DNA sensor, cGAS is activated upon DNA binding and catalyzes the production of cGAMP, the second messenger that initiates downstream STING signaling. We therefore first examined whether X‐DNA can interact with cGAS and promote cGAMP production (Figure 1a). X‐DNA, a four‐way Holliday junction assembled from four DNA strands (Table S1), typically exists in two conformations in solution: the open X and the stacked X. The open X predominates under physiological conditions, whereas ionic or protein binding can induce the stacked X form, which often serves as a preferred substrate for dimeric resolvases and recognition proteins [29, 30]. Based on this structural feature, we propose that cGAS binding may induce a conformational transition of X‐DNA that facilitates its activation. To examine this, we employed atomic force microscopy (AFM) to visualize conformational changes of X30 before and after cGAS binding, using the inter‐arm distance as an indicator of conformational switching (Figure 1c). The results showed that free X30 molecules exhibited a typical cross‐shaped open X structure, whereas co‐incubation with cGAS led to a significant reduction in arm‐to‐arm distance, resulting in a more compact morphology. This conformational contraction indicates that cGAS binding induces a transition of X‐DNA from the open X to the stacked X, providing direct visual evidence of their interaction.

**FIGURE 1:**
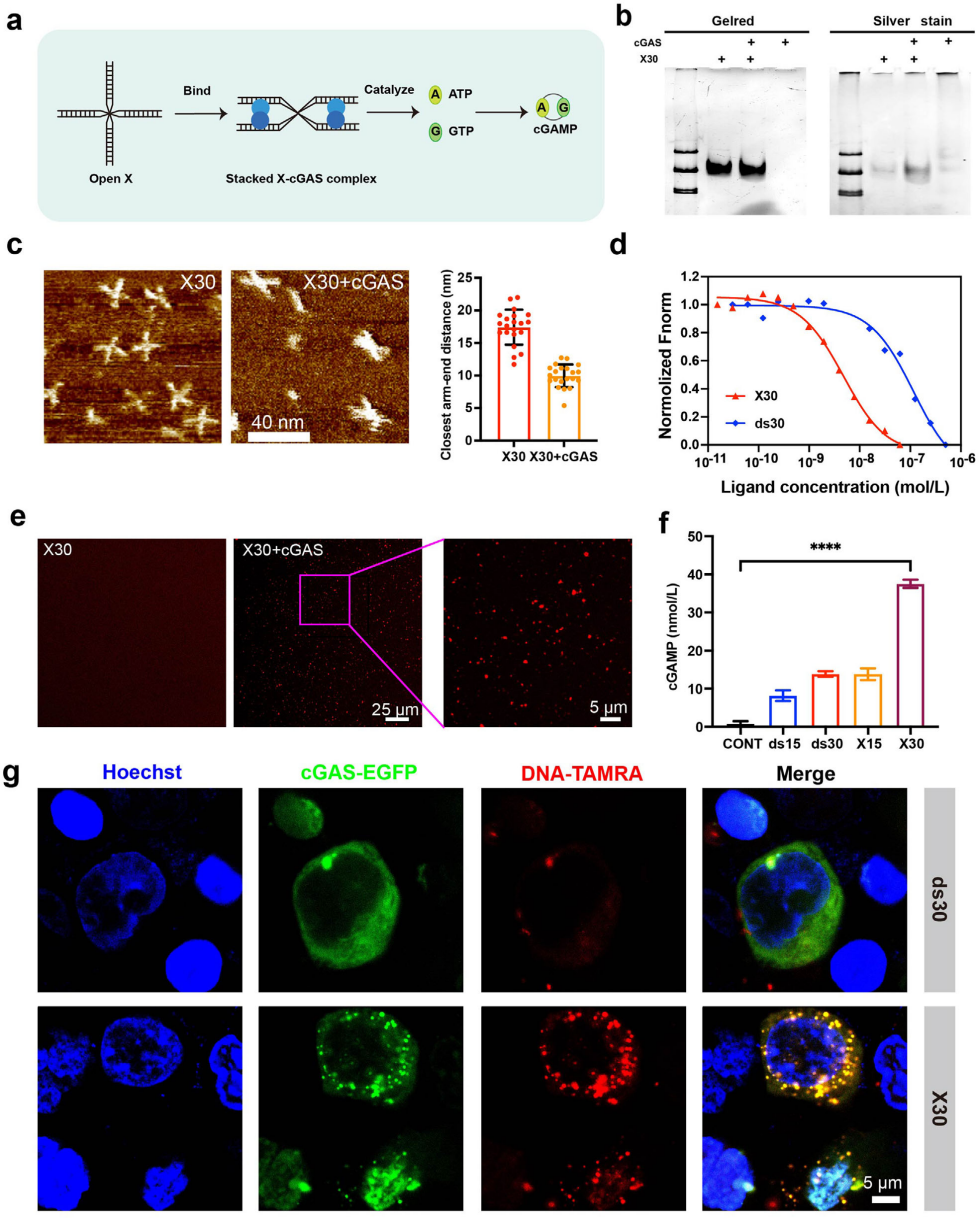
Interaction between X‐DNA and cGAS. (a) Schematic illustration of cGAS binding‐induced conformational transition of X‐DNA and subsequent cGAMP production. (b) Native PAGE analysis of X30 co‐incubated with cGAS. Nucleic acids were stained with GelRed, and proteins were visualized using silver staining. Uncropped images are provided in Figure S1. (c) AFM images of X30 before and after incubation with cGAS, with statistical analysis of inter‐arm distances. Quantitative data are shown as mean ± SEM (*n* = 21). (d) Binding curves of X30 and ds30 with cGAS measured by MST. X30 (*K_D_*: 3.2 ± 0.7 nM); ds30 (*K_D_*: 106.0 ± 18.2 nM). (e) Confocal fluorescence images of fluorescently labeled X30 alone and after incubation with cGAS. (f) cGAMP levels in BMDCs after 2 h stimulation with ds15, ds30, X15, or X30. Data are shown as mean ± SEM (*n* = 3). One‐way ANOVA followed by Tukey's multiple‐comparisons test was performed. ****p < 0.0001. (g) Confocal images of ds30/X30 co‐localized with cGAS‐EGFP in HEK293T cells.

To verify complex formation and quantify binding strength, native PAGE and microscale thermophoresis (MST) were performed. GelRed staining was used to visualize nucleic acids, while silver staining was used to detect proteins. Silver staining revealed that the cGAS signal redistributed from the top of the gel to the position corresponding to X30 after co‐incubation, indicating that cGAS co‐migrates with X‐DNA under native conditions (Figure 1b; Figure S1). MST measurements further revealed a strong enhancement in binding affinity compared with linear DNA of the same length. This result supports the formation of X‐DNA‐cGAS complexes. The binding curve of X30 with cGAS was markedly shifted to the left, yielding a dissociation constant (*K_D_*) of 3.2 ± 0.7 nM, whereas double‐stranded DNA (ds30) exhibited a *K_D_* of 106.0 ± 18.2 nM, approximately a 33‐fold difference (Figure 1d). This affinity enhancement corresponds to an energetic gain of ∼8.7 kJ·mol^−1^, indicating that the interaction between cGAS and X‐DNA is thermodynamically much more favorable.

Following co‐incubation with cGAS, fluorescently labeled X30 exhibited pronounced clustering (Figure 1e). In contrast, X30 alone showed a predominantly diffuse fluorescence distribution, whereas the presence of cGAS resulted in the formation of numerous discrete fluorescent puncta. These observations suggest local co‐enrichment and clustering of cGAS and X30. We next examined whether this enhanced interaction leads to distinct intracellular behavior and functional activation. In HEK293T cells overexpressing cGAS‐EGFP, co‐incubation with either ds30 or X30 was performed to assess the intracellular distribution of cGAS (Figure 1g). Following ds30 treatment, cGAS signals were evenly dispersed throughout the cytoplasm and showed weak colocalization with ds30. In contrast, cells treated with X30 exhibited a marked redistribution of cGAS‐EGFP, characterized by the emergence of multiple punctate or granule‐like enrichment regions that strongly colocalized with X30. This transition from a diffuse to a punctate pattern indicates that X‐DNA induces local enrichment and spatial reorganization of cGAS within the cytoplasm. Notably, previous studies have reported that cytosolic DNA can trigger liquid‐liquid phase separation of cGAS to form droplet‐like condensates [28]. Here, the punctate pattern indicates local enrichment and clustering of cGAS, but whether this represents phase separation remains unclear. Finally, intracellular cGAMP production was measured in BMDCs to evaluate functional output (Figure 1f). X‐DNA constructs induced significantly higher cGAMP levels than their linear counterparts, with X30 showing the strongest activation.

### 2.2 X‐DNA Topology Facilitates Multivalent cGAS Assembly

We sought to elucidate the molecular mechanism by which X‐DNA activates cGAS. *Andreeva* et al. reported that the cGAS_1_‐DNA_1_ complex appears to be catalytically inactive, whereas the cGAS_2_‐DNA_2_ complex is intrinsically unstable and requires sufficiently long DNA to assemble into a stable ladder‐like cGAS_2n_‐DNA_2_ network (n ≥ 2) (Figure 2a). cGAS preferentially recognizes structured DNA messengers or DNA associated with residual nuclear proteins, rather than naked DNA [27]. Increasing evidence indicates that the spacing between adjacent DNA duplexes in cGAS‐DNA assemblies and viral particles is approximately 1 nm [23]. These observations suggest that DNA spatial organization may critically influence the stability and cooperativity of cGAS‐DNA assembly. In this context, X‐DNA provides a multibranched nucleic acid topology that spatially constrains adjacent double‐stranded DNA segments while retaining conformational flexibility—distinct from rigid DNA origami. We hypothesize that the Holliday junction core in X‐DNA maintains adjacent DNA arms within a favorable spatial range, thereby reducing the instability of the cGAS_2_‐DNA_2_ complex (Figure 2b). Moreover, the intrinsic symmetry of X‐DNA may further promote multivalent cGAS assembly, reminiscent of the ladder‐like networks observed previously [27].

**FIGURE 2:**
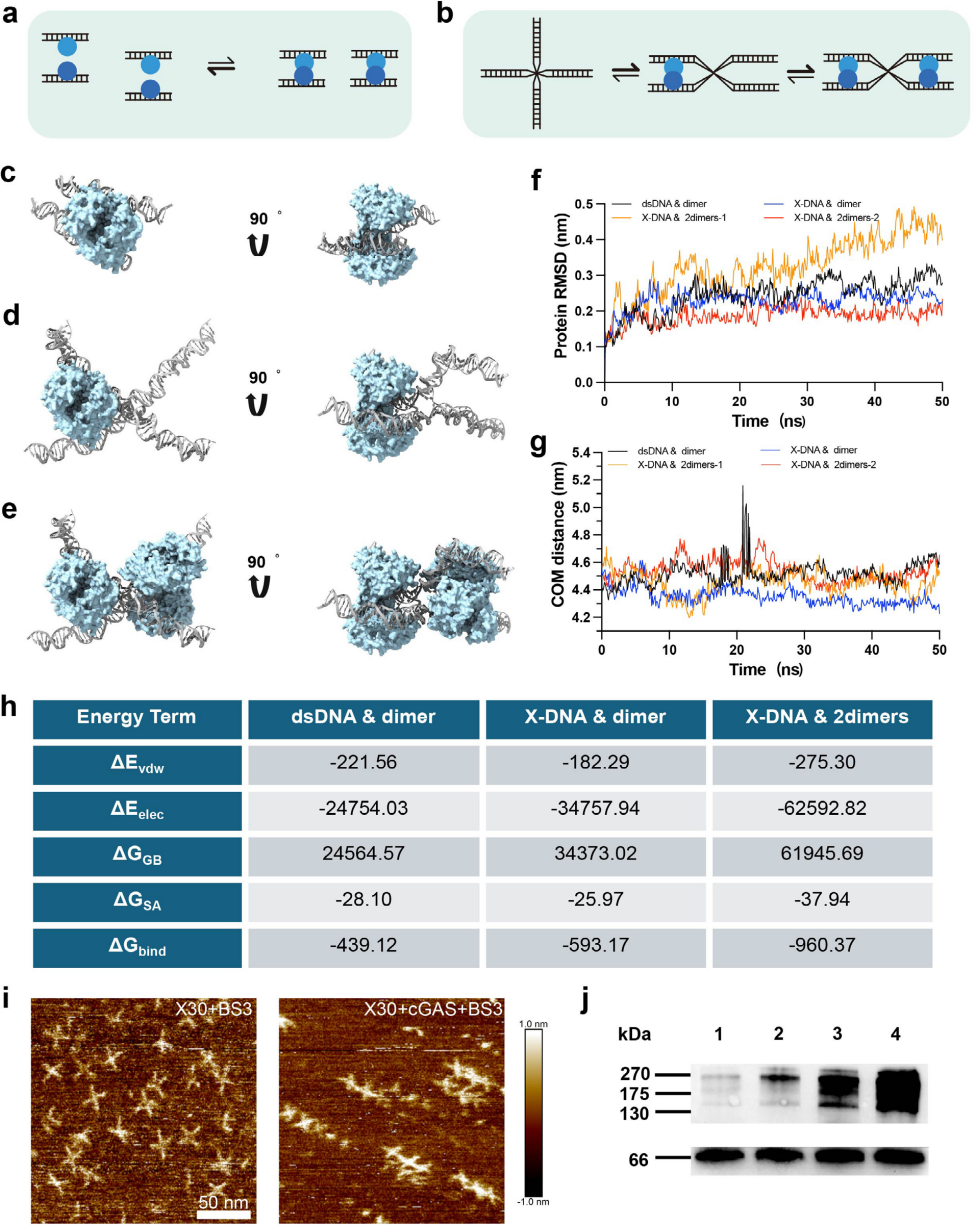
(a, b) Conceptual model of DNA‐structure dependence. Activation requires a cGAS_2_‐DNA_2_ synapse. With linear dsDNA (a), the synapse forms only transiently and is unstable. X‐DNA (b) presents four arms that stabilize cGAS dimers and offers multiple, spatially arranged binding sites for additional dimers. (c) Docking model of a 30‐bp dsDNA (ds30) with a cGAS dimer. (d) Docking model of X30 with a single cGAS dimer. (e) Docking model of X30 simultaneously engaging two cGAS dimers. The structural modeling was conducted via the HDOCK server (http://hdock.phys.hust.edu.cn/), using the cGAS crystal structure (PDB ID: 4LEZ) as the binding template for the docking simulations. (f) Protein RMSD over 50 ns MD trajectories. (g) Distance between the centers of mass of the two cGAS monomers within the dimer during the simulations. (h) MM/GBSA binding free‐energy results (ΔE_vdw_, ΔE_ele_, ΔG_GB_, ΔG_SA_, and ΔG_bind_). (i) AFM images of X30 treated with BS3 and X30‐cGAS complexes after BS3‐mediated chemical crosslinking. (j) Western blot analysis of chemically crosslinked cGAS complexes. Lane 1, cGAS; lane 2, cGAS + BS3; lane 3, cGAS + ds30 + BS3; lane 4, cGAS + X30 + BS3. Uncropped images are provided in Figure S2.

To verify our hypothesis, we first employed an integrated docking‐molecular dynamics (MD) approach to evaluate the feasibility of our design. Molecular docking was used to predict potential binding conformations, followed by detailed analysis based on molecular dynamics simulations. (Detailed procedures for molecular docking and molecular dynamics simulations are provided in the Supporting Information.) The cGAS dimer was used as the target, while ds30 and X30 served as ligands for docking. The docking results are shown in Figure 2c,d. The docked conformation of the ds30‐cGAS_2_ complex was highly consistent with previously reported crystal structures, confirming the reliability of this approach. As expected, the docking of X‐DNA with cGAS revealed that the cGAS dimer was positioned at the inter‐arm angle of X‐DNA, with a relative orientation similar to that observed in the ds30‐cGAS complex. The two arms of X‐DNA wrapped around the dimeric interface, exposing the catalytic pocket responsible for cGAMP synthesis, indicating that the binding mode of X‐DNA to cGAS follows the canonical cGAS‐dsDNA assembly pattern. To further assess the binding capacity of X‐DNA, we docked a second cGAS dimer onto the preformed X‐DNA‐cGAS_2_ complex. Interestingly, an additional binding site was observed at the symmetric position (Figure 2e). Unexpectedly, the two cGAS dimers adopted a head‐to‐head configuration, consistent with the multivalent cGAS‐dsDNA assembly pattern reported previously [27].

We next assessed the stability of the modeled complexes through 50 ns molecular dynamics simulations. The RMSD values of the cGAS dimers remained within 5 Å throughout the trajectories, indicating that all dimers were relatively stable, with the X‐DNA group showing better overall stability than the dsDNA group (Figure 2f). Interestingly, in the presence of a second dimer, one of the cGAS dimers exhibited smaller fluctuations, suggesting enhanced structural stability. We further analyzed the centroid distance between the two cGAS monomers within each dimer. Notably, only the dsDNA group displayed abrupt changes in inter‐monomer distance, which may correspond to the intrinsic instability of the cGAS_2_‐DNA_2_ complex described earlier (Figure 2g). In contrast, X‐DNA effectively alleviated this instability. Importantly, binding free‐energy analysis using the MM/GBSA method revealed that X‐DNA‐dimer complexes exhibited lower ΔG_bind_ values than dsDNA‐dimer complexes, indicating stronger binding and greater assembly stability. Moreover, the ΔG_bind_ value of the X‐DNA‐2dimers complex was significantly lower, suggesting that X‐DNA more readily promotes the multivalent assembly of cGAS dimers (Figure 2h).

To further obtain experimental evidence for cGAS oligomerization, we performed chemical crosslinking to stabilize cGAS molecules that were in close spatial proximity within the DNA‐cGAS complexes. In the AFM images, neighboring X30 molecules were observed to be connected through the crosslinked cGAS proteins (Figure 2i). Because BS3 crosslinks proximal proteins rather than DNA, this crosslinking‐dependent connection supports the presence of a DNA‐cGAS‐cGAS‐DNA assembly, which is consistent with the well‐established cGAS_2_‐DNA_2_ complex demonstrated in structural and biochemical studies. Western blot analysis of the crosslinked products further showed prominent high‐molecular‐weight cGAS bands in the X30 group, mainly distributed between approximately 130 and 270 kDa (Figure 2j; Figure S2), corresponding approximately to dimeric, trimeric, and tetrameric cGAS species. The appearance of these high‐molecular‐weight species provides biochemical evidence for the formation of cGAS oligomeric assemblies in the presence of X30. Together, these results experimentally support the proposed model in which the multibranched topology of X‐DNA facilitates cGAS_2_‐DNA_2_ assembly and further promotes higher‐order cGAS organization.

Consistent with the length‐dependent activation mechanism of cGAS by DNA, we further investigated the effect of arm length in X‐DNA (X15, X20, and X25) (Figures S3–S6). Notably, X15 did not exhibit a favorable binding mode with the cGAS dimer, suggesting that insufficient arm length limits effective engagement with the dimer interface. In contrast, X20 showed a docking configuration with a single cGAS dimer comparable to that of X30; however, it failed to accommodate a second dimer in a stable head‐to‐head arrangement, indicating that its arm length is still insufficient to support multivalent assembly. Increasing the arm length to X25 partially restored this capability, allowing simultaneous engagement of two cGAS dimers in a configuration similar to X30. These results collectively suggest that there exists a critical arm‐length threshold for X‐DNA to stabilize higher‐order cGAS assemblies. Only when the arm length is sufficient can X‐DNA effectively support the cooperative binding of multiple dimers, thereby promoting the formation of stable cGAS signaling complexes.

### 2.3 X‐DNA Activates the cGAS‐STING Pathway in DCs

After entering DCs, X‐DNA was expected to engage cytosolic cGAS, promote cGAMP production, activate the STING‐dependent TBK1/IRF3 signaling cascade, and ultimately induce type‐I interferon secretion and DC maturation (Figure 3a). To isolate topology effects at a fixed dsDNA content, X30 was compared with a mixture of four separate 30‐bp dsDNA fragments (ds30), while X15 was compared with a mixture of four separate 15‐bp dsDNA fragments (ds15). In each comparison, DNA doses were normalized according to total base‐pair content. Accordingly, when expressed as molar concentration, the concentration of the linear dsDNA was fourfold that of the corresponding X‐DNA, thereby maintaining an equivalent total amount of ds DNA. Both X30 and ds30 were delivered using the same Lipofectamine 3000 transfection system under matched DNA‐dose conditions to maintain consistent experimental conditions.

**FIGURE 3:**
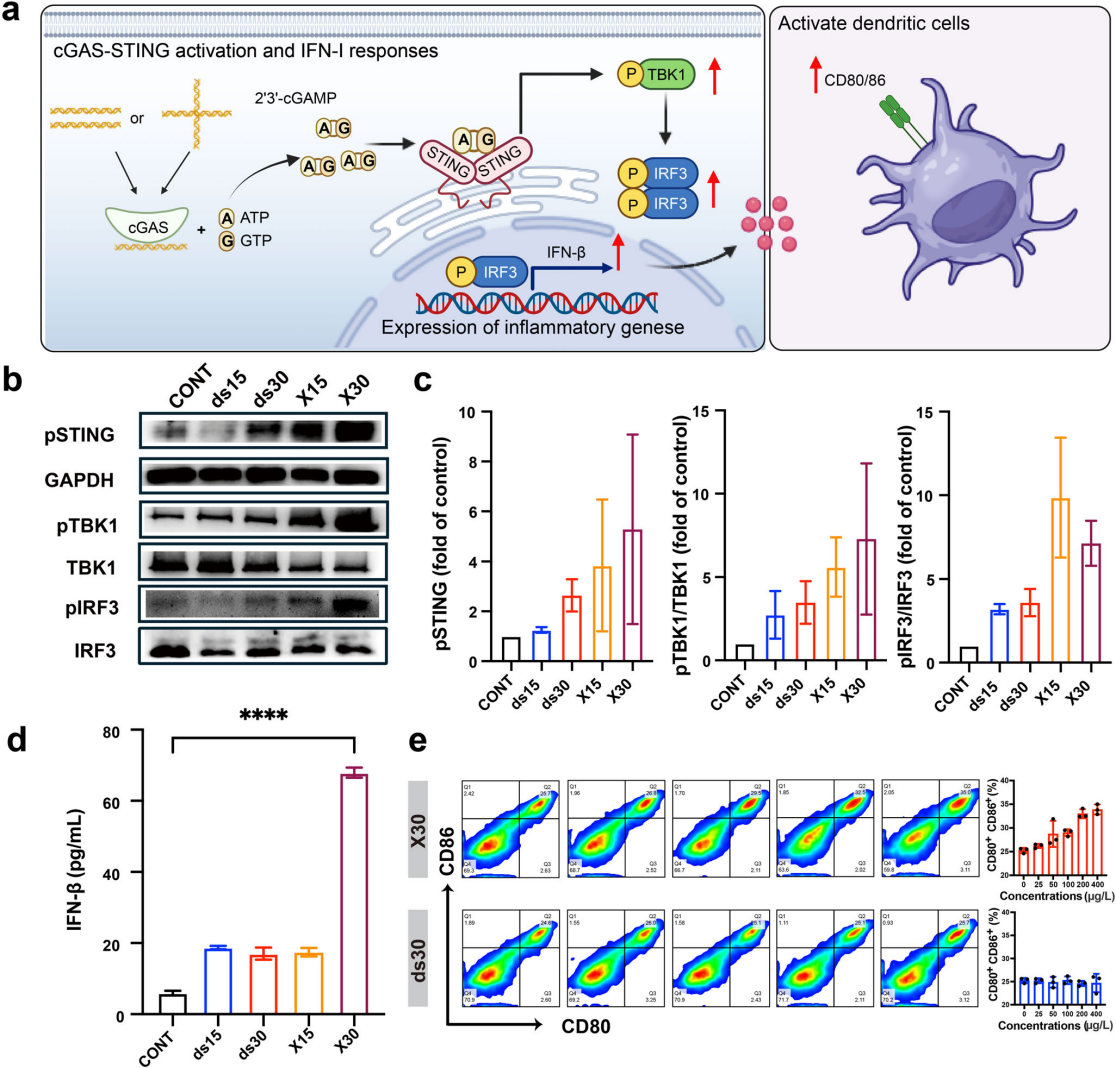
(a) Schematic representation of dsDNA/X‐DNA activating BMDCs via the cGAS‐STING pathway. (b) Western blot analysis showing protein expression levels in BMDCs. Uncropped images are provided in Figure S11. (c) Quantification of the Western blot results. Quantitative data are shown as mean ± SEM (*n* = 3). (d) IFN‐β levels in BMDC supernatants after 24 h stimulation with ds15, ds30, X15, or X30. Data are shown as mean ± SEM (*n* = 3). One‐way ANOVA followed by Tukey's multiple‐comparisons test was performed. ****p < 0.0001. (e) CD80 and CD86 expression in BMDCs after 24 h stimulation with ds30 or X30 at 0.25, 0.5, 1, 2, and 4 µg/mL. Quantitative data are shown as mean ± SEM (*n* = 3).

In mouse bone‐marrow‐derived dendritic cells (BMDCs), we found that X‐DNA activated the cGAS‐STING pathway more strongly than the length‐matched dsDNA controls (Figure 3b–d). STING, a downstream adaptor of cGAS, increased in abundance, and the ratios of phosphorylated to total TBK1 and IRF3 were elevated, with the largest changes observed for X30 (Figure 3b,c; Figure S11). In line with this pathway engagement, culture supernatants contained higher concentrations of IFN‐β after stimulation with X‐DNA than with dsDNA, and X30 elicited the strongest response (Figure 3d). Building on prior evidence that cGAS‐STING activation in dendritic cells induces type‐I interferon, upregulates CD80/CD86, and enhances cross‐presentation and T‐cell priming [31, 32], we assessed whether X30 functionally activates BMDCs. Flow cytometry showed clear dose‐dependent increases in CD80 and CD86 after 24 h exposure to X30 at 0.25–4 µg mL^−1^, whereas ds30 produced little change across the same range (Figure 3e).

The cGAS dependence of this response was then examined in a genetic loss‐of‐function setting using WT and *Cgas^−^/^−^* MC38 cells. X30‐induced *Ifnb* and *Cxcl10* expression was markedly reduced in *Cgas^−^/^−^* cells, whereas cGAMP remained strongly active (Figure S7). Together with the STING, TBK1, and IRF3 activation observed in BMDCs, these results support a cGAS‐dependent mechanism for X‐DNA‐induced type I interferon signaling.

We hypothesize that the observed differences arise from length‐ and topology‐dependent requirements for cGAS engagement in cells. Although a 12‐bp dsDNA fragment is sufficient to span one cGAS binding site [20], the shortest duplex known to form a cGAS_2_‐DNA_2_ complex is at least 16 bp [33]. For very short duplexes, a sufficiently high copy number is still required to reach an avidity threshold [27]. Prior studies also indicate that cGAS activation depends on dsDNA mechanical flexibility [34]. In our constructs, the X‐DNA arm length sets the effective bending compliance and spacing of parallel duplexes. X15 arms are likely constrained by mechanical flexibility and therefore fail to support productive assembly of cGAS dimers in BMDCs. By contrast, X30 provides longer, more compliant arms that better accommodate cGAS dimer bridging and oligomerization. In addition, X‐DNA showed an arm‐length‐dependent activation pattern, with longer arms more effectively supporting productive cGAS engagement and DC activation (Figure S8a). Taken together, these results suggest that the enhanced activity of X30 arises from the combined effects of sufficient arm length and X‐shaped topology. Sufficient duplex length enables productive cGAS engagement, while the pre‐organized multivalent architecture of X‐DNA increases the local density and spatial organization of DNA‐binding sites, thereby favoring cooperative cGAS assembly. Nevertheless, differences in cellular uptake, endosomal escape, and intracellular stability may still exist between different DNA structures. These potential differences represent a limitation of the present study and warrant further investigation in future work.

### 2.4 X‐DNA‐Based Peptide Vaccine Induces APC Activation and Antigen Presentation to Trigger T Cell Immunity

Having established that X‐DNA binds cGAS with high affinity and triggers potent activation of the cGAS‐STING pathway, we next explored whether this property could be harnessed for rational vaccine design. By using X‐DNA as an immunostimulatory scaffold, we conjugated the OVA peptide to X‐1 via click chemistry and subsequently assembled the structure with complementary strands to generate the X‐DNA‐based peptide vaccine (OVA‐X30) (Figure 4a). This modular strategy allows the antigen to be precisely positioned on an immunostimulatory backbone, which may facilitate both efficient innate sensing and antigen presentation.

**FIGURE 4:**
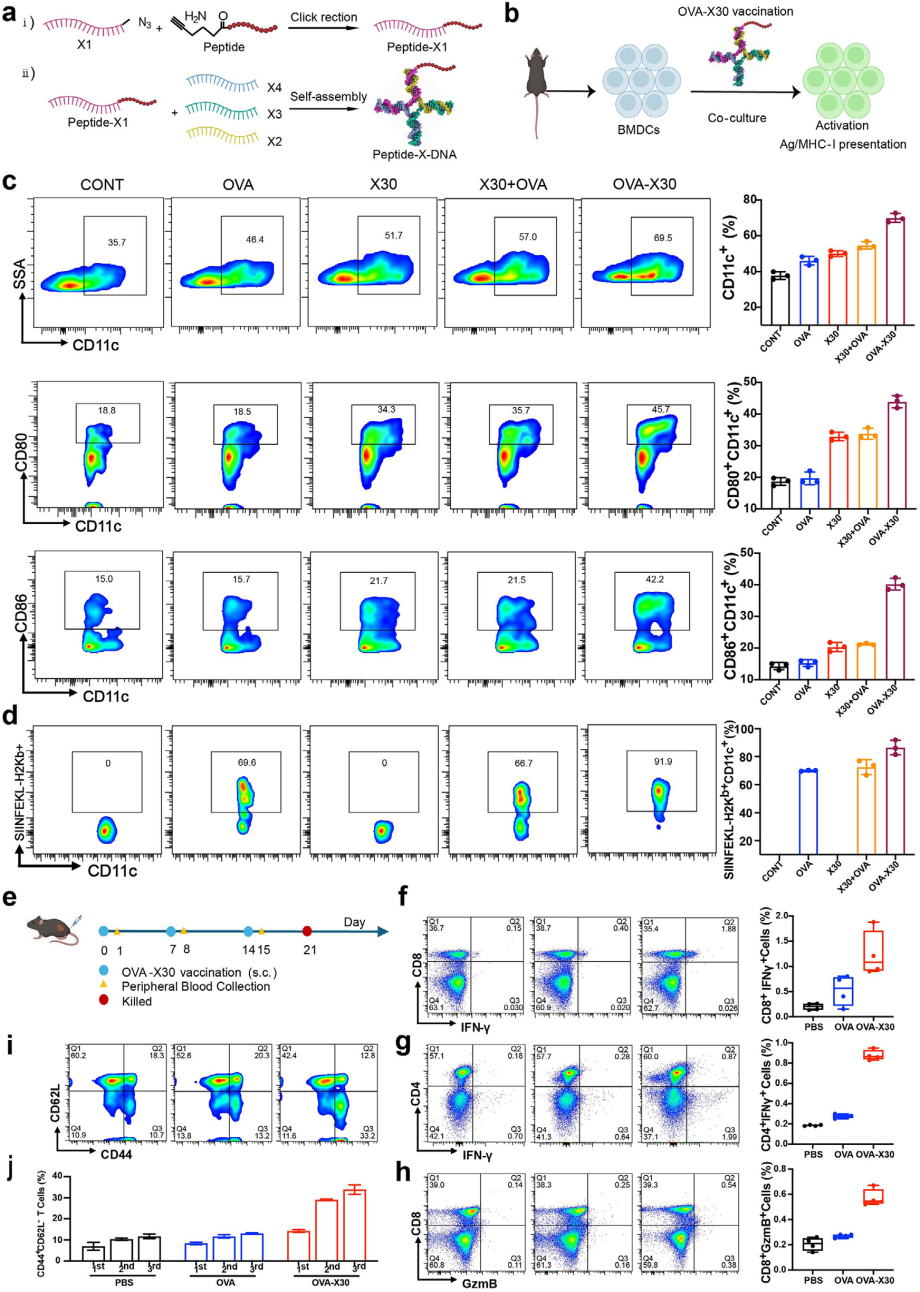
Delivery of OVA‐X30 vaccine to APCs and triggered T cell immunity. (a) Flowchart for constructing the X‐DNA‐based peptide vaccine. (b) Schematic illustration of OVA‐X30 vaccine reconfiguration in DCs, subsequent DC activation, and antigen presentation. (c) Expression of CD11c, CD80, and CD86 in BMDCs after treatment with PBS (CONT), OVA, X30, a mixture of X30 and OVA, or the OVA‐X30 formulation. Quantitative data are shown as mean ± SEM (*n* = 3). (d) Quantification of SIINFEKL‐presenting BMDCs after treatment with the same formulations. Quantitative data are shown as mean ± SEM (*n* = 3). (e) Schematic illustration of the immunization and sampling schedule. Mice were subcutaneously injected in the inguinal region with the vaccine on days 0, 7, and 14. Peripheral blood was collected on days 1, 8, and 15 to monitor the immune response. On day 21, mice were sacrificed, and spleens were harvested for immune response evaluation. (f–h) Flow cytometry analysis of antigen‐specific T cell responses. Splenocytes were isolated and stimulated ex vivo with SIINFEKL peptide. Shown are the frequencies of IFN‐γ‐producing CD8^+^ T cells (f), IFN‐γ‐producing CD4^+^ T cells (g), and Granzyme B‐producing CD8^+^ T cells (h) in mice immunized with PBS, OVA, or the OVA‐X30 vaccine. Quantitative data are shown as mean ± SEM (*n* = 3). (i, j) Analysis of peripheral blood effector T cell responses. Peripheral blood was collected after each immunization, and CD44^+^CD62L^−^ effector T cells were analyzed by flow cytometry. (i). The frequencies of effector T cells were quantified at each time point (j) in mice immunized with PBS, OVA, or the OVA‐X30 vaccine. Quantitative data are shown as mean ± SEM (*n* = 3).

We first examined the ability of OVA‐X30 to activate DCs in vitro (Figure 4b; Figure S8). Remarkably, compared to free OVA, X30 alone, or their simple mixture, the OVA‐X30 formulation induced a pronounced upregulation of the costimulatory molecules CD80 and CD86 (Figure 4c), hallmarks of DC maturation. In line with this, a markedly higher proportion of SIINFEKL‐H‐2Kb^+^CD11c^+^ DCs was observed in the OVA‐X30 group than in all other treatments (Figure 4d), indicating efficient antigen processing and MHC I presentation—a prerequisite for CD8^+^ T cell priming. This effect likely reflects the synergistic action of the X‐DNA scaffold and the conjugated peptide: the X‐DNA moiety provides strong innate stimulation through cGAS recognition, while the covalently linked antigen ensures its concurrent processing and presentation.

Encouraged by these in vitro findings, we next evaluated the immunostimulatory potential of OVA‐X30 in vivo. Mice were immunized subcutaneously on days 0, 7, and 14, with blood collected after each dose and spleens harvested on day 21 (Figure 4e). Consistent with the in vitro data, OVA‐X30 vaccination elicited strong antigen‐specific T cell responses: significantly more IFN‐γ‐producing CD8^+^ and CD4^+^ T cells, as well as Granzyme B‐producing CD8^+^ T cells, were detected in the spleen compared with the PBS and OVA groups (Figure 4f–h). The simultaneous induction of IFN‐γ^+^ CD8^+^ and IFN‐γ^+^ CD4^+^ T cells represents a critical benchmark for evaluating vaccine‐induced cellular immunity. IFN‐γ^+^ CD8^+^ T cells (cytotoxic T lymphocytes, CTLs) reflect the vaccine's ability to elicit potent cytolytic responses, which are essential for clearing infected or malignant cells. In parallel, IFN‐γ^+^ CD4^+^ T cells indicate a robust Th1‐type helper response, which plays indispensable roles in sustaining CTL proliferation and function, promoting high‐affinity antibody production, and supporting the formation of long‐term immune memory. Moreover, the induction of Granzyme B^+^ CD8^+^ T cells indicates that OVA‐X30 vaccination not only promotes IFN‐γ production but also drives functional cytotoxic differentiation, endowing T cells with the machinery required for effective target cell killing. This combination of Th1 helper activity, CTL activation, and effector differentiation is a hallmark of vaccines capable of eliciting both rapid and durable cellular immunity. Supporting these findings, peripheral blood analysis showed a progressive expansion of CD44^+^CD62L^−^ effector T cells over the course of immunization, peaking after the third dose (Figure 4i,j), consistent with strong priming and effector maturation.

### 2.5 Tumor Prevention and Tumor Treatment

A key goal of therapeutic cancer vaccination is not only to induce robust immune responses but also to achieve effective therapeutic control and tumor prevention in vivo. To evaluate the protective and therapeutic efficacy of the OVA‐X30 vaccine, we performed both tumor prevention and treatment studies using the B16‐OVA melanoma model, which is widely regarded as a stringent preclinical system for testing T cell‐based vaccines.

We first examined the therapeutic efficacy of OVA‐X30 in mice bearing established tumors. Mice were inoculated subcutaneously with B16‐OVA tumor cells and subsequently vaccinated with OVA‐X30 on days 4, 11, and 18 (Figure 5a). OVA‐X30 treatment markedly inhibited tumor growth compared to the PBS and OVA groups, as shown by both individual and mean tumor growth curves (Figure 5c,d). Mice treated with OVA‐X30 showed significantly prolonged survival compared with those in the PBS and OVA groups (Figure 5b). These results demonstrate that the X‐DNA‐based peptide vaccine can effectively mobilize antitumor immunity to control established tumors.

**FIGURE 5:**
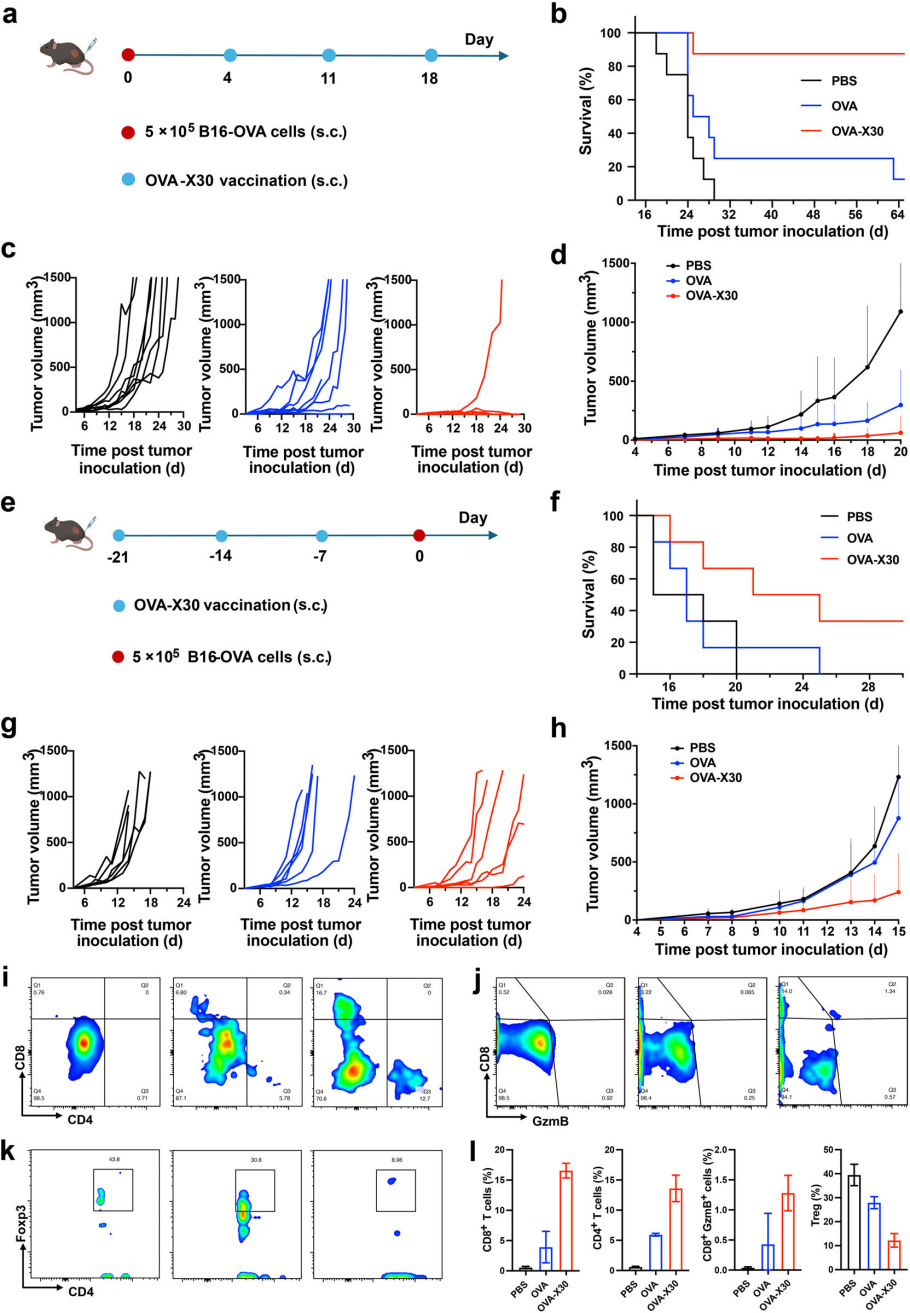
(a) Timeline of the tumor treatment experiment. Mice were first inoculated subcutaneously with 5 × 10^5^ B16‐OVA tumor cells on day 0, followed by subcutaneous vaccination with OVA‐X30 on days 4, 11, and 18. (b) Survival curves of mice after tumor challenge. c Individual tumor growth curves in each group. Data are shown as mean ± SEM (*n* = 6–8 mice per group). (d) Mean tumor volume over time in each group. (e) Timeline of the tumor prevention experiment. Mice were immunized subcutaneously with the OVA‐X30 vaccine on days 0, 7, and 14, followed by subcutaneous inoculation of 5 × 10^5^ B16‐OVA tumor cells on day 21. (f) Survival curves. (g) Individual tumor growth curves. (h) Mean tumor volume after treatment. Data are shown as mean ± SEM (*n* = 6–8 mice per group). (i) Representative flow cytometry plots showing the proportions of CD4^+^ and CD8^+^ T cells among tumor‐infiltrating CD3^+^ T cells. (j) Representative flow cytometry plots showing GzmB expression in tumor‐infiltrating CD8^+^ T cells. (k) Representative flow cytometry plots showing Foxp3^+^ regulatory T (Treg) cells among tumor‐infiltrating CD4^+^ T cells. (l) Quantification of CD8^+^ T cells, CD4^+^ T cells, CD8^+^GzmB^+^ T cells, and Treg cells in tumors. Quantitative data are shown as mean ± SEM (*n* = 3).

We next evaluated the preventive efficacy of OVA‐X30 using a prophylactic vaccination model. Mice were immunized on days 0, 7, and 14 and challenged with B16‐OVA tumor cells one week after the final immunization (Figure 5e). In this setting, OVA‐X30 vaccination conferred strong protection against tumor development, as reflected by delayed or absent tumor growth in most mice (Figure 5g,h; Figure S10a) and significantly improved survival compared with control groups (Figure 5f). The body weight of all groups remained stable throughout the experiments, indicating good tolerability (Figures S9 and S10b). To further characterize the tumor immune microenvironment, tumor‐infiltrating T cells were analyzed by flow cytometry. OVA‐X30 markedly increased the proportions of both CD8^+^ and CD4^+^ T cells among tumor‐infiltrating CD3^+^ T cells compared with the PBS and OVA groups (Figure 5i,l). The proportion of CD8^+^GzmB^+^ T cells was also increased in the OVA‐X30 group (Figure 5j,l), whereas the proportion of Foxp3^+^ regulatory T (Treg) cells among CD4^+^ T cells was substantially reduced (Figure 5k,l). These results indicate that OVA‐X30 promotes intratumoral T‐cell responses while reducing immunosuppressive Treg cells.

From the perspective of cancer vaccine evaluation, therapeutic and preventive models provide complementary evidence of efficacy. Therapeutic models test whether a vaccine can generate effector T cells capable of controlling established disease, while prevention models assess the induction of durable immune memory capable of rejecting future tumor challenges. The potent antitumor effects observed in both settings highlight the strong cellular immunity and protective potential elicited by the OVA‐X30 vaccine. These findings, together with the robust T cell responses described earlier, support the utility of X‐DNA‐based peptide vaccines as versatile platforms for both cancer treatment and prevention.

## 3 Conclusions

This study establishes a topology‐driven strategy for innate immune activation and demonstrates that X‐DNA, a four‐arm Holliday‐junction framework, serves as a structural and functional modulator of the cGAS‐STING pathway. Through combined computational and experimental analyses, we show that the multi‐arm geometry of X‐DNA stabilizes cGAS_2_‐DNA_2_ synapses, facilitates multivalent assembly, and induces a conformational transition from the open X to the stacked X, thereby enhancing cGAS binding and activation. At the cellular level, X‐DNA promotes the spatial reorganization and punctate enrichment of cGAS, suggesting that signal amplification may be associated with enhanced cGAS clustering.

Functionally, X‐DNA acts as a potent nucleic acid adjuvant that boosts IFN‐β production and dendritic cell maturation, outperforming linear dsDNA of equivalent length. When conjugated with the OVA antigenic peptide, the resulting OVA‐X30 vaccine achieves spatial coupling between antigen presentation and innate immune stimulation, eliciting strong Th1‐biased immune responses and potent antitumor efficacy in both prophylactic and therapeutic melanoma models.

Collectively, these findings reveal that programmable nucleic acid topology can be harnessed to precisely modulate cGAS recognition and innate immune activation, offering a versatile platform for the rational design of next‐generation structural adjuvants and DNA‐scaffolded peptide vaccines.

## 4 Experimental Section

### 4.1 Construction and Characterization of X‐DNA‐Based Peptide Vaccines

To construct the X‐DNA‐based OVA peptide vaccine, OVA peptide (SIINFEKLRRG‐propargylglycine) was conjugated to the 5′‐azide‐modified X30‐1 strand (Table S1) via copper(I)‐catalyzed azide‐alkyne cycloaddition (CuAAC). Briefly, the propargylglycine‐modified peptide (200 µM, 50 µL) was combined with the azide‐modified X30‐1 strand (200 µM, 25 µL) and DMSO (25 µL), followed by addition of Cu^2+^/TBTA (10 mM, 25 µL) and freshly prepared sodium ascorbate (10 mM, 25 µL). The reaction was carried out overnight at 25°C, and the products were purified by 12% denaturing PAGE to obtain the DNA‐OVA conjugate. This conjugated strand was assembled with X30‐2, X30‐3, and X30‐4 in PBS, heated to 95°C for 5 min, and slowly cooled to 4°C, yielding the final framework X‐DNA‐based peptide vaccine, OVA‐X30, which was verified by PAGE and characterized by AFM.

### 4.2 Native PAGE and Silver Staining

X30, cGAS, and the pre‐incubated X30‐cGAS mixture were analyzed using 10% native polyacrylamide gel electrophoresis (PAGE) under non‐denaturing conditions. After electrophoresis, nucleic acids were visualized by GelRed staining. For protein visualization, parallel gels were subjected to silver staining using a commercial silver staining kit according to the manufacturer's instructions.

### 4.3 Atomic Force Microscopy (AFM)

AFM imaging was performed in liquid using a MultiMode 8 atomic force microscope (Bruker, USA) operated in ScanAsyst in Fluid mode. Samples were deposited onto freshly cleaved mica and imaged directly in liquid. Images were acquired at a scan rate of approximately 1 Hz with a resolution of 512 × 512 pixels and analyzed using NanoScope software.

### 4.4 Microscale Thermophoresis (MST)

The binding affinities of ds30 and X30 for recombinant cGAS were determined separately by microscale thermophoresis (MST) using a Monolith NT.115 Pico instrument (NanoTemper Technologies, Germany). X30 or ds30 was used as the target at a fixed concentration of 50 nm, while cGAS served as the ligand and was serially diluted from 125 nM to approximately 15.3 pM. Samples were loaded into Monolith NT.115 capillaries and measured using Pico‐RED excitation with the excitation power set to 100% and the MST power set to medium. Binding curves were fitted using a model based on the law of mass action to determine *K_D_* values.

### 4.5 Chemical Crosslinking of cGAS‐X‐DNA Complexes

Chemical crosslinking was performed in a total reaction volume of 20 µL containing cGAS and X30 or ds30 at a cGAS:DNA molar ratio of 4:1. The reaction buffer contained 20 mM HEPES (pH 7.5), 100 mM NaCl, and 5 mM MgCl_2_. cGAS and X30 were pre‐incubated at room temperature for 15 min, followed by addition of freshly prepared BS3 to a final concentration of 0.5 mM. Crosslinking was allowed to proceed for 30 min at room temperature and was terminated by addition of 1 µL of 1 M Tris‐HCl (pH 7.5), followed by incubation for an additional 15 min at room temperature. The crosslinked products were subsequently analyzed by SDS‐PAGE and western blotting using an anti‐cGAS antibody.

### 4.6 cGAMP Quantification

Intracellular cGAMP levels were quantified using a cGAMP ELISA kit (MEIABIO, Shanghai, China) according to the manufacturer's instructions. BMDCs were treated with the indicated DNA samples for 2 h, after which the cells were collected and lysed. The resulting cell lysates were subjected to ELISA, and the absorbance was measured using a microplate reader. cGAMP concentrations were calculated from the corresponding standard curve.

### 4.7 Animals

C57BL/6 mice (6 weeks old) were purchased from Shanghai Laboratory Animal Center (China). All animals were housed with a 12 h light/dark cycle at 25°C, 40% relative humidity and food and water ad libitum. All animal studies were performed in accordance with ARRIVE guidelines. All animal experiments were approved by the Experimental Animal Welfare Ethics Committee of Renji Hospital, Shanghai Jiao Tong University School of Medicine (Approval No. RJ2023‐060A).

### 4.8 Cell Culture

Murine BMDCs were isolated from femurs and tibiae of C57BL/6 mice and maintained in RPMI‐1640 medium supplemented with 10% FBS, 100 units per mL penicillin and 100 units per mL streptomycin at 37°C in 5% CO_2_. Recombinant mouse GM‐CSF (20 ng ml^−1^) and IL‐4 (10 ng ml^−1^) were added to induce cell differentiation. The medium was half‐replaced every 2 days. On the sixth day of culture, the medium was gently pipetted; immature BMDCs that were suspended in the medium and loosely adhered to the flask were all collected for further use.

The mouse melanoma B16‐OVA cells and MC38 colon adenocarcinoma cells were maintained in DMEM medium supplemented with 10% fetal bovine serum (FBS), 100 units per mL penicillin, and 100 units per mL streptomycin at 37°C in 5% CO_2_. *Cgas^−/−^* MC38 cells were kindly provided by Prof. Jun Gui (Renji Hospital, Shanghai Jiao Tong University School of Medicine) and maintained under the same culture conditions as wild‐type MC38 cells. All cell lines were routinely tested and confirmed to be free of mycoplasma contamination.

### 4.9 Western Blot Analysis

Immature BMDCs (1 × 10^6^ per ml) were plated onto non‐tissue‐culture‐treated 24‐well dishes (Corning) and stimulated with nucleic acids ds15, ds30, X15, or X30 at a final concentration of 4 µg/mL using Lipofectamine 3000. After incubation at 37°C with 5% CO_2_ for 24 h, cells were harvested and lysed in RIPA buffer supplemented with protease and phosphatase inhibitors. Protein concentrations were determined by BCA assay, and equal amounts of protein (20–30 µg) were separated by SDS‐PAGE and transferred onto PVDF membranes (Millipore). Membranes were blocked with 5% nonfat milk in TBST for 1 h at room temperature and incubated overnight at 4°C with primary antibodies against GAPDH, STING, TBK1, p‐TBK1, IRF3, and p‐IRF3. After washing, membranes were incubated with HRP‐conjugated secondary antibodies for 1 h at room temperature, and signals were detected using enhanced chemiluminescence (ECL) reagents and imaged with a chemiluminescence detection system.

### 4.10 IFN‐I Expression

Immature BMDCs (1 × 10^6^ per mL) were plated onto non‐tissue‐culture‐treated 24‐well dishes (Corning) and stimulated with nucleic acids ds15, ds30, X15, or X30 at a final concentration of 4 µg/mL, delivered using Lipofectamine 3000. Cells were incubated at 37°C with 5% CO_2_ for 24 h, and the supernatants were collected to determine the concentration of IFN‐β by ELISA.

### 4.11 DC Activation and Antigen Presentation

Immature BMDCs (1 × 10^6^ per mL) were plated onto non‐tissue‐culture‐treated 24‐well dishes and stimulated for 24 h at 37°C, 5% CO_2_ with ds30 or X30 delivered by Lipofectamine 3000, at final nucleic acid doses of 0.25, 0.5, 1, 2, or 4 µg/mL. After incubation, cells were stained with antibodies against CD80 and CD86 and analyzed by flow cytometry.

For vaccine evaluation, BMDCs were treated for 24 h with one of five conditions: cont, OVA, X30, X30+OVA, or OVA‐X30. Following stimulation, cells were stained for CD11c, CD80, CD86, and SIINFEKL‐presenting populations, and analyzed by flow cytometry.

### 4.12 Molecular Dynamics (MD) Simulations

Three cGAS‐DNA complexes were modeled: (i) dsDNA bound to a cGAS dimer (dsDNA & dimer), (ii) X‐DNA bound to a cGAS dimer (X‐DNA & dimer), and (iii) X‐DNA engaging two cGAS dimers (X‐DNA & 2 dimers). Proteins were protonated at pH 7.0, missing side chains were rebuilt, and termini were capped. Systems were parameterized with the CHARMM36 family (CHARMM36m for protein, CHARMM36 for nucleic acids) and solvated in CHARMM‐modified TIP3P water in a dodecahedral box extending ≥10 Å from any solute atom. Na^+^/Cl^−^ ions (CHARMM parameters) were added to neutralize and reach 150 mM. After 5000 steps of steepest‐descent plus conjugate‐gradient minimization, we performed restrained equilibration at 300 K: NVT for 200 ps and NPT for 1 ns (heavy‐atom position restraints 1000 kJ·mol^−1^·nm^−2^, then released in stages). Production MD was run with GROMACS 2025 for 3 × 50 ns per system (independent seeds), 2 fs time step, LINCS constraints on bonds to H, PME electrostatics (real‐space cutoff 1.2 nm, grid spacing ∼0.12 nm), LJ force‐switch 1.0→1.2 nm for van der Waals, v‐rescale thermostat (300 K) and Parrinello‐Rahman barostat (1 bar). Coordinates were saved every 10 ps. Analyses included protein Cα RMSD and DNA‐protein center‐of‐mass distances over time.

### 4.13 MM/GBSA Calculations

For each snapshot, the binding free energy was omputed as

$$\Delta G_{\mathrm{bind}}=G_{\mathrm{complex}}-\left(G_{\mathrm{receptor}}+G_{\mathrm{ligand}}\right)=\Delta E_{\mathrm{MM}}+\Delta G_{\mathrm{solv}}-T\Delta S$$

with

$$\Delta E_{\mathrm{MM}}=\Delta E_{\mathrm{vdw}}+\Delta E_{\mathrm{ele}},\;\Delta G_{\mathrm{solv}}=\Delta G_{\mathrm{GB}}+\Delta G_{\mathrm{SA}}$$

Here Δ*G_bind_* is the MM/GBSA binding free energy; Δ*E_vdw_* and Δ*E_ele_* are the gas‐phase van der Waals and Coulomb terms, respectively; Δ*G_GB_* is the polar solvation contribution from the GB‐Neck2 model (internal dielectric = 1.0; solvent dielectric = 80.0; igb = 8, i.e., GB‐Neck2); and Δ*G_SA_* is the non‐polar solvation term estimated from solvent‐accessible surface area,

$$\Delta G_{\mathrm{SA}}=\gamma \Delta \mathrm{SASA}+b\left(\gamma \approx 0.0072\;\mathrm{kcal}\;\mathrm{mo}l^{1}\;{\overset{\circ }{A}}^{-2},\;b\approx 0\right)$$

Unless stated otherwise, entropic contributions (*T*Δ*S*, e.g., normal‐mode/quasi‐harmonic) were not included; thus the reported Δ*G_bind_* corresponds to

$$\Delta E_{\mathrm{vdw}}+\Delta E_{\mathrm{ele}}+\Delta G_{\mathrm{GB}}+\Delta G_{\mathrm{SA}}$$

### 4.14 Fluorescence Microscopy

HEK293T cells were grown on chambered cover glass to ∼70% confluence and transfected with a mouse cGAS‐EGFP plasmid using a standard transfection reagent. Stable cGAS‐overexpressing cells were generated by antibiotic selection and expansion, then reseeded to a proper density for imaging. Cells were incubated with fluorescently labeled X‐DNA (50 nM in culture medium) at 37°C for 6 h, rinsed three times with PBS, and returned to culture medium at 37°C for 30 min before imaging. Live‐cell images were acquired on a confocal fluorescence microscope using a 60× oil objective.

### 4.15 Splenocyte Isolation and Intracellular Cytokine Staining After Vaccination

Mice were immunized on days 0, 7, and 14 with the indicated vaccine at 1,500 pmol per mouse per dose. On day 21, mice were euthanized and spleens were harvested. Each spleen was minced and homogenized in 250 µL digestion medium (collagenase I 45 U µL^−1^, DNase I 25 U µL^−1^, hyaluronidase 30 U µL^−1^), transferred to a 15 mL tube containing 5–10 mL digestion medium, filtered through a 70 µm strainer, and washed once with PBS. Cells were pelleted (300 × *g*, 5 min, 4°C), resuspended in 5 mL RBC lysis buffer (BioLegend) for 5 min on ice, pelleted again (300 × *g*, 5 min, 4°C), and washed twice with PBS, then seeded in RPMI 1640. For flow cytometry, cells were stained first for surface markers (CD3, CD8, CD4; total volume 100 µL, 30 min, dark, 4°C), washed twice with PBS, then fixed and permeabilized (eBioscience Foxp3/Transcription Factor kit) and stained for intracellular IFN‐γ and granzyme B (GzmB). Data were acquired on a Beckman Coulter flow cytometer and analyzed with FlowJo.

### 4.16 Analysis of Memory T Cells

One day after each vaccine dose, ∼200 µL peripheral blood was collected and processed for PBMC isolation as follows: blood was transferred to a 15 mL tube containing 10 mL cold PBS, mixed, and centrifuged at 600 × *g* for 5 min at room temperature. The pellet was resuspended in 2 mL ACK lysis buffer (NH_4_Cl 0.15 M, KHCO_3_ 10 mM, EDTA 0.1 mM; pH 7.2) for RBC lysis, quenched by adding 10 mL cold PBS, and centrifuged again at 600 × g for 5 min. PBMCs were washed in FACS staining buffer and counted. Cells were then stained with anti‐mouse CD8α, CD44, and CD62L antibodies (0.25 µg each in 100 µL staining buffer, 20 min on ice), washed twice, and analyzed by flow cytometry. Effector memory T cells (Tem) were defined as CD8^+^CD44^+^CD62L^−^.

### 4.17 Tumor Prevention Experiments

C57BL/6 mice (6 weeks) were randomly assigned to three groups: PBS, OVA, and OVA‐X30. Mice were immunized subcutaneously on days 0, 7, and 14 with the indicated formulation (1,500 pmol per mouse per dose for OVA and OVA‐X30; PBS group received equal‐volume PBS). On day 21, mice were challenged by subcutaneous inoculation of B16‐OVA cells (5 × 10^5^ in PBS) into the right flank. Tumor growth was monitored three times per week with digital calipers, and volumes were calculated as 0.5 × length × width^2^. Mice were euthanized when tumor volumes reached 1,500 mm^3^ or per humane endpoints.

### 4.18 Tumor Immune Analysis

Tumor tissues were collected, minced into small pieces, and enzymatically digested to obtain single‐cell suspensions. The suspensions were filtered through a 70 µm cell strainer and subjected to red blood cell lysis. Cells were then stained with antibodies against CD45, CD3, CD4, and CD8 to evaluate tumor‐infiltrating T‐cell populations. For intracellular cytokine analysis, cells were fixed and permeabilized and subsequently stained for Foxp3, IFN‐γ and Granzyme B. Samples were analyzed by flow cytometry, and the frequencies of tumor‐infiltrating immune cell populations were quantified using FlowJo software.

### 4.19 Tumor Therapy Experiments

C57BL/6 mice (6 weeks) were inoculated subcutaneously with B16‐OVA cells (5 × 10^5^ in PBS) into the right flank on day 0, then randomly assigned to PBS, OVA, or OVA‐X30 groups. Therapeutic vaccinations began when tumors reached ∼50 mm^3^ (day 4 post‐inoculation) and were administered subcutaneously on days 4, 11, and 18 (1,500 pmol per mouse per dose for OVA and OVA‐X30; PBS group received equal‐volume PBS). Tumor growth was measured three times per week. Mice were euthanized when tumor volumes reached 1,500 mm^3^ or per humane endpoints.

### 4.20 Statistical Analysis

Statistical analysis was performed using Microsoft Excel and Prism 8.0 (GraphPad). The survival difference was assessed by log‐rank (Mantel‐Cox) test. Differences among the experimental groups were assessed using two‐tailed unpaired t‐test, one‐way or two‐way analysis of variance (ANOVA) with Tukey's multiple‐comparisons test. A difference was considered significant if *p* < 0.05 (*p < 0.05, **p < 0.01, ***p < 0.001, ****p < 0.0001). For cell‐based assays, biological replicates represent independent experiments performed using separately prepared cell samples. For animal experiments, each mouse was considered an independent biological replicate.

## Author Contributions

J.L., Y.Y. and S.W. designed the study; J.L. performed the experiments; J.L. analyzed the data; and J.L., Y.Y. and S.W. wrote the paper. All the authors have read and approved the final version of the manuscript.

## Conflicts of Interest

The authors declare no conflicts of interest.

## Supporting information




**Supporting File**: advs78563‐sup‐0001‐SuppMat.docx.

## Data Availability

The data that support the findings of this study are available from the corresponding author upon reasonable request.
