# Supplementary material for: Topology‐Driven X‐Shaped DNA as a cGAS Stimulator to Promote Dendritic Cell Activation and Antitumor Vaccine Efficacy
Source: Adv Sci (Weinh). 2026 Oct 29:e78563. Online ahead of print. doi: 10.1002/advs.78563 (PMC13720384; doi:10.1002/advs.78563)
Supplement: Supplementary file 1 [file ADVS-9999-e78563-s001.docx]

**Supporting Information**

**Topology-Driven X-Shaped DNA as a cGAS Stimulator to Promote Dendritic Cell Activation and Antitumor Vaccine Efficacy**

Jiabei Li^1^, Xuan Wang^2^, Dali Wei^2^, Zhejie Chen^2^, Ziyi Wang^2^, Qing Yu^2^, Yu Yang^2^*, Shengpeng Wang^1^*

^1^State Key Laboratory of Mechanism and Quality of Chinese Medicine, Institute of Chinese Medical Sciences, University of Macau, Macao SAR 999078, China

^2^Institute of Molecular Medicine (IMM), Renji Hospital, School of Medicine, Shanghai Jiao Tong University, Shanghai 200240, China.

**Table of Contents**

**Experimental Section	3**

**Table S1. Strands for synthesizing X-DNA	4**

**Figure S1. Uncropped images corresponding to Figure 1b	5**

**Figure S2. Uncropped images corresponding to Figure 2j	6**

**Figure S3. Characterization of X-DNA	6**

**Figure S4. Docking model of X15 with a single cGAS dimer	6**

**Figure S5. Docking model of X20 with cGAS dimer(s)	7**

**Figure S6. Docking model of X25 with cGAS dimer(s)	7**

**Figure S7. Real-time PCR analysis of *Ifnb* and *Cxcl10* expression in WT and *Cgas*^⁻/⁻^ MC38 tumor cells treated with Lipofectamine (CONT), ds30, X30, or cGAMP for 24 h.	8**

**Figure S8. Activation and antigen-presentation analysis of BMDCs	9**

**Figure S9. Mouse body weight during tumor treatment experiment	10**

**Figure S10. Tumor weight (a) and mouse body weight (b) during tumor prevention experiment	10**

**Figure S11. Uncropped Western blot images corresponding to Figure 3b	11**

**Experimental Section**

**Materials**

All ODNs used were purchased from Huzhou Hema Biotechnology Co., Ltd. (Huzhou, China). Recombinant cGAS proteins (MedChemExpress, Cat# HY-P71597, HY-P72337, and HY-P703401) were obtained from MedChemExpress (MCE). The primary antibodies against cGAS (E5V3W) (Cell Signaling Technology, Cat. No. 79978S), phospho-STING (Ser365) (Cell Signaling Technology, Cat# 72971T), TBK1/NAK (Cell Signaling Technology, Cat# 3504T), phospho-TBK1/NAK (Ser172) (Cell Signaling Technology, Cat# 5483T), IRF-3 (Cell Signaling Technology, Cat# 4302T), phospho-IRF-3 (Ser396) (Cell Signaling Technology, Cat# 29047T), and GAPDH (Beyotime, Cat# AF0006) were purchased from the indicated suppliers. HRP-labeled goat anti-rabbit IgG (Beyotime, Cat# A0208) and HRP-labeled goat anti-mouse IgG (H+L) (Beyotime, Cat# A0216) were used as secondary antibodies for western blotting. Flow cytometry antibodies against APC-CD11c (BD Biosciences, Cat# 550261), FITC-CD80 (BD Biosciences, Cat# 561954), PE-CD86 (BD Biosciences, Cat# 553692), FITC-CD3 (BD Biosciences, Cat# 558091), APC-CD4 (BD Biosciences, Cat# 553051), PE-CD8 (BD Biosciences, Cat# 553032), PerCP-CD45 (BD Biosciences, Cat# 557235), APC-IFN-γ (BD Biosciences, Cat# 554413), PE-FOXP3 (Invitrogen, Cat#12-5773-82) and PE-Cy7-Granzyme B (Invitrogen, Cat# 25-8898-82) were purchased from the indicated suppliers. The silver staining kit (Beyotime, Cat# P0017S) was obtained from Beyotime Biotechnology. Lipofectamine 3000 was purchased from Thermo Fisher Scientific. RNA isolater Total RNA Extraction Reagent (Vazyme, Cat. No. R401-01), HiScript III RT SuperMix for qPCR (Vazyme, Cat. No. R323-01), and ChamQ Universal SYBR qPCR Master Mix (Vazyme, Cat. No. Q711) were purchased from Vazyme Biotech Co., Ltd. (Nanjing, China). DMEM, RPMI-1640, fetal bovine serum (FBS), penicillin, and streptomycin were obtained from Gibco (Thermo Fisher Scientific, USA).

**Fabrication of framework nucleic acids**

To assemble the X-DNA, the designed DNA strands listed in **Table S1** were mixed in an equimolar ratio in PBS to a final strand concentration of 2 μM. Specifically, strands X15-1, X15-2, X15-3, and X15-4 were combined to assemble X15, and strands X30-1, X30-2, X30-3, and X30-4 were combined to assemble X30. Mixtures were heated to 95 °C for 5 min and then slowly cooled to 4 °C. The resulting framework nucleic acids were purified by high-performance liquid chromatography (HPLC), stored at 4 °C.

**Table S1**. Strands for synthesizing X-DNA:

|  | Sequences (5’-3’) |
| --- | --- |
| X15-1 | CAGTTGAGACGAACACTAAGTCTGAAATTT |
| X15-2 | AAATTTCAGACTTAGGAATGTTCGACATGC |
| X15-3 | GCATGTCGAACATTCCTGAATTGCACGGGC |
| X15-4 | GCCCGTGCAATTCAGTGTTCGTCTCAACTG |
| X30-1 | GACTCAGGTGCTGACTTTGGCATTCGACCAGGAGATATCGCGTTCAGCTATGCCCACCTG |
| X30-2 | CAGGTGGGCATAGCTGAACGCGATATCTCCACGTCAGTCCGGTTTCCCACACGGGACGGC |
| X30-3 | GCCGTCCCGTGTGGGAAACCGGACTGACGTTATGTGTTCTGTGCGGCCTGCCGTCCCGTG |
| X30-4 | CACGGGACGGCAGGCCGCACAGAACACATATGGTCGAATGCCAAAGTCAGCACCTGAGTC |
| 5’-azide-modified X30-1 | N_3_-GACTCAGGTGCTGACTTTGGCATTCGACCAGGAGATATCGCGTTCAGCTATGCCCACCTG |

**Real-time PCR (RT-PCR)**

WT and *Cgas^⁻/⁻^* MC38 cells were treated with Lipofectamine 3000 alone (CONT), ds30, X30, or cGAMP for 24 h. ds30 and X30 were delivered using Lipofectamine 3000 under matched DNA-dose conditions. After treatment, total RNA was extracted and reverse-transcribed into cDNA. Real-time PCR was performed using a SYBR Green-based quantitative PCR system. The relative mRNA expression levels of *Ifnb* and *Cxcl10* were normalized to *Gapdh* and calculated using the 2^⁻ΔΔCt^ method. Experiments were performed with three biological replicates.

The primer sequences were as follows: *Gapdh*, forward： 5’-AGCTTGTCATCAACGGGAAG-3’, reverse: 5’-TTTGATGTTAGTGGGGTCTCG-3’; *Ifnb*, forward: 5’-AGCTCCAAGAAAGGACGAACA-3’, reverse: 5’-GCCCTGTAGGTGAGGTTGAT-3’; *Cxcl10*, forward: 5’-CCAAGTGCTGCCGTCATTTTC-3’, reverse: 5’-GGCTCGCAGGGATGATTTCAA-3’.

**Figure S1.** Uncropped images corresponding to **Figure 1b**.

**Figure S2.** Uncropped images corresponding to **Figure 2j**. Membranes were cut into strips before antibody incubation.

**Figure S3. a** AFM images of X-DNA. Scale bars, 80 nm. **b** Native PAGE to demonstrate the formation of X-DNA. Lanes 1-4 show X15 assembly: Lane 1: X15-1; Lane 2: X15-1 + X15-2; Lane 3: X15-1 + X15-2 + X15-3; Lane 4: X15-1 + X15-2 + X15-3 + X15-4 (X15). Lanes 5-8 show X30 assembly: Lane 5: X30-1; Lane 6: X30-1 + X30-2; Lane 7: X30-1 + X30-2 + X30-3; Lane 8: X30-1 + X30-2 + X30-3 + X30-4 (X30). **c** Hydrodynamic size of X-DNA measured by DLS.

**Figure S4.** Docking model of X15 with a single cGAS dimer.

**Figure S5.** **a** Docking model of X20 with a single cGAS dimer. **b** Docking model of X20 simultaneously engaging two cGAS dimers.

**Figure S6.** **a** Docking model of X25 with a single cGAS dimer. **b** Docking model of X25 simultaneously engaging two cGAS dimers.

**Figure S7.** Real-time PCR analysis of *Ifnb* and *Cxcl10* expression in WT and *Cgas^⁻/⁻^* MC38 tumor cells treated with Lipofectamine (CONT), ds30, X30, or cGAMP for 24 h. Data are shown as mean±SEM (n = 3). Two-way ANOVA followed by Tukey’s multiple-comparisons test was performed. *P < 0.05; **P < 0.01; ***P < 0.001; ****P < 0.0001; ns, not significant.

**Figure S8.** **a** Expression of CD80, and CD86 in BMDCs. Data are shown as mean±SEM (n = 3). **b, c** Quantification of SIINFEKL-presenting BMDCs after treatment with the same formulations. Data are shown as mean±SEM (n = 3).

**Figure S9.** Mouse body weight during tumor treatment experiment. Data are shown as mean±SEM (n=6-8 mice per group).

**Figure S10.** Tumor weight (a) and mouse body weight (b) during tumor prevention experiment. Data are shown as mean±SEM (n=6-8 mice per group).

**Figure S11.** **Uncropped Western blot images corresponding to Figure 3b.** Membranes were cut into strips according to the molecular weights of the target proteins before antibody incubation. GAPDH and the target proteins were detected on separate membranes.
